# Detection of Aflatoxins in Different Matrices and Food-Chain Positions

**DOI:** 10.3389/fmicb.2020.01916

**Published:** 2020-08-14

**Authors:** Gabriella Miklós, Cserne Angeli, Árpád Ambrus, Attila Nagy, Valéria Kardos, Andrea Zentai, Kata Kerekes, Zsuzsa Farkas, Ákos Jóźwiak, Tibor Bartók

**Affiliations:** ^1^Székesfehérvár Regional Food Chain Laboratory, National Food Chain Safety Office, Székesfehérvár, Hungary; ^2^Fumizol Ltd., Szeged, Hungary; ^3^University of Debrecen Doctoral School of Nutrition and Food Sciences, Debrecen, Hungary; ^4^Food Chain Safety Laboratory Directorate, National Food Chain Safety Office, Budapest, Hungary; ^5^System Management and Supervision Directorate, National Food Chain Safety Office, Budapest, Hungary; ^6^Digital Food Institute, University of Veterinary Medicine Budapest, Budapest, Hungary

**Keywords:** aflatoxins, LOD, LOQ, limits, extraction, clean-up, analysis, detection

## Abstract

Aflatoxins, produced mainly by filamentous fungi *Aspergillus flavus* and *Aspergillus parasiticus*, are one of the most carcinogenic compounds that have adverse health effects on both humans and animals consuming contaminated food and feed, respectively. Aflatoxin B1 (AFB1) and aflatoxin B2 (AFB2) as well as aflatoxin G1(AFG1) and aflatoxin G2 (AFG2) occur in the contaminated foods and feed. In the case of dairy ruminants, after the consumption of feed contaminated with aflatoxins, aflatoxin metabolites [aflatoxin M1 (AFM1) and aflatoxin M2 (AFM2)] may appear in milk. Because of the health risk and the official maximum limits of aflatoxins, there is a need for application of fast and accurate testing methods. At present, there are several analytical methods applied in practice for determination of aflatoxins. The aim of this review is to provide a guide that summarizes worldwide aflatoxin regulations and analytical methods for determination of aflatoxins in different food and feed matrices, that helps in the decision to choose the most appropriate method that meets the practical requirements of fast and sensitive control of their contamination. Analytical options are outlined from the simplest and fastest methods with the smallest instrument requirements, through separation methods, to the latest hyphenated techniques.

## Introduction

Mycotoxins are secondary metabolites of filamentous fungi and their presence indicates biological contamination. These compounds may enter the human and animal bodies directly by the consumption of contaminated agricultural products or ready-to-eat products or indirectly through the consumption of animal products (mainly milk, eggs, and offal), deriving from animals that consumed contaminated feed (Adányi, 2013).

Aflatoxins are the first known mycotoxin group, described as a result of turkey “X” disease in the 1960s (Blount, 1961; Wannop, 1961). Mycotoxin research has begun worldwide from that time on.

**FIGURE 1:**
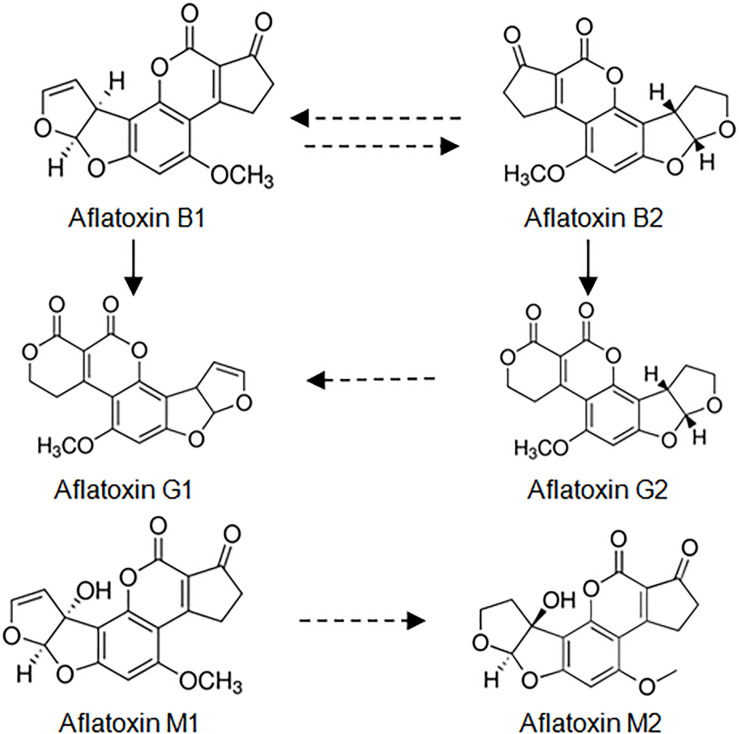
Transformation of aflatoxins.

More than ten types of aflatoxins exist naturally, of which AFB1 is the most toxic. AFB1 and AFB2, AFG1, and AFG2 occur in the contaminated feed. AFM1 and AFM2 are present in ruminant milk after the digestion of feed contaminated by AFB1 and AFB2. In order to analyze aflatoxins, various analytical methods are required. Transformation of aflatoxins can be seen in Figure 1.

There is a wealth of scientific information with respect to aflatoxins and their acute and chronic effects and numerous research groups have worked on this topic recently. According to Web of Science, there are nearly 16,000 publications since 1975 to this day in connection with aflatoxins, of which over 7,000 have been published in the last decade. These numbers and legal restrictions across the world regarding the highly carcinogenic aflatoxins indicate the importance of the topic.

This publication gives a complex and transparent summary of the regulatory environment and the diverse measurement techniques of aflatoxins from rapid methods through seemingly simple separation techniques to complex hyphenated techniques. Sample preparation methods associated with the different measurement techniques are also covered.

## Analytical Expectations

Free trade of food and feed is getting more and more common around the world. In order to keep the product flow under control, there is a need for harmonized regulation and control systems both in exporting and importing countries. Because of this, many countries have already established common regulations and maximum levels for different contaminants, including aflatoxins. Nonetheless, some non-community countries (Table 1) have their own maximum levels for aflatoxins. There are different maximum permitted levels around the world mainly regarding AFB1 and aflatoxins total (AFT) (AFB1, AFB2, AFG1, and AFG2) for food and feed and AFM1 for milk and milk products. Consequently, it is important to be aware of these regulations, among others, for selecting appropriate analytical methods to verify the necessary compliance. Examples for the different regulations regarding aflatoxin levels are shown in Table 1.

**TABLE 1:** Worldwide aflatoxin regulations, allowed maximum levels.

Communities	Countries	Organization	Reference of regulation	Aflatoxin B1 (μg/kg) (food)	Total Aflatoxin (μg/kg) (food)	AflatoxinM1 (μg/kg)	Aflatoxin B1 (μg/kg) (feed)	Total Aflatoxin (μg/kg) (feed)
African Union (AU)	South Africa	South Africa Department of Health		5	10	x	x	x
ASEAN (Association of Southeast Asian Nations)	Brunei	Department of Health Services, Ministry of Health		0	0	0	x	x
	Cambodia			x	x	x	x	x
	Democratic Republic of Laos, Myanmar			x	x	x	x	x
	Indonesia	National Agency of Drug and Food Control (NADFC)		15	0.5–5	0.5–5	x	20–50
	Malaysia	Food Safety and Quality Division, Ministry of Health Malaysia		0.1	5–35	0.025–0.5	x	x
	Philippines	Department of Agriculture		10	10–50	0.5	20	x
	Singapore	Food Regulations	Singapore Government, 2002	0.1–5	5	0.025–0.5	x	x
	Thailand	Bureau of Quality and Safety of Food (BQSF)		x	15–50	x	x	x
	Vietnam	National Institute for Food Control		0.1–12	4–15	0.025–0.5	x	x
CODEX				x	15	x	x	x
Codex GCC (Gulf Cooperation Council)	Bahrain, Yemen, Kuwait, Oman, Qatar, Saudi Arabia, United Arab Emirates			5–12	0.05–20	0.05	x	x
EU (European Union)		European Food Safety Authority	Food: Commission Regulation *European Commission* (EC) No 1881/2006 Feed: EU Directive 2002/32 and EU Recommendation 2006/576/EC	0.1–12	4–15	0.025–0.050	5–20	x
MERCOSUR (Mercado Común del Sur) (Southern Common Market)	Argentina, Paraguay, Uruguay, Venezuela (suspended since 2016)			x	20	x	x	x
	Brazil			0.5–2.5	1–20	0.5–2.5	x	x
USA (United States of America)		Food and Drug Administration	US FDA, 2019	x	20	0.5	x	x
	Algeria			8		x	x	x
	Australia, New-Zealand	Australian New Zealand Food Standards Code (ANZFA)		x	15	0.02	x	x
	Bosnia and Herzegovina			8–12	10–15	x	x	x
	Canada	Canadian Food Inspection Agency		x	15	x	x	x
	China		USDA, 2014	0.5–20	only for: Chinese medicine: Chen pi, suan zao ren, jiang can, pang da hai, tao ren: 10	0.5–20	≤10–≤50	0.5
	Egypt			0.1–12	4–15	x	x	x
	India	APEDA (Agricultural and Processed Food Products Export Development Authority)		x	10–15	x	x	x
	Japan	Food Safety Commission; Feed: MAFF (Ministry of Agriculture, Forestry and Fisheries)	USDA, 2010	10	10	0.5	10–20	10–1000
	Korea			0.1–10	x	0.1–10	15	0.5
	Mexico			x	20	x	x	x
	Nigeria	National Agency For Food And Drug Administration And Control (NAFDAC)		20	x	x	x	x
	Peru	Codex		x	15	x	x	x
	Russia			5	x	x	x	x
	Turkey			8–12	12–15	x	x	x
	Ukraine			8–12	10–15	x	x	x

As Table 1 shows, the regulatory environment varies greatly in different areas. Therefore, high performance and sensitivity of the analytical methods are not always necessary in the case of controlling the compliance with legal limits. Nonetheless, product control has to be carried out in economically underdeveloped countries as well, where more sophisticated analytical techniques and instruments are rarely available. However, in some cases, where the legal limits are lower (e.g., in the European Union or ASEAN countries), more sensitive methods have to be used (Williams et al., 2004).

In Supplementary Table 1, methods for aflatoxin measurement, which will be discussed later, are summarized.

## Sample Preparation Methods

Mycotoxins are toxic chemical compounds with low molecular weight (MW < 1000), and due to their diverse chemical structure, there exists no single standard technique for their analysis and/or detection (Turner et al., 2009).

Most of the methods used are based on appropriate extraction and clean-up. Sample preparation is one of the most important steps in the determination of mycotoxins. It may add up to two-thirds of the time of the full analysis and could significantly affect the accuracy and precision of the results. The most commonly used clean-up methods applied in aflatoxin analysis are liquid–liquid extraction (LLE), solid-phase extraction (SPE) and QuEChERS (Quick, Easy, Cheap, Effective, Rugged, and Safe) methods. In addition, there are a number of other extraction methods in the literature that are less widely used in routine analysis at present.

### Extraction and Clean-Up Methods

#### Liquid–Liquid Extraction (LLE)

This is a simple and cheap method for the extraction of aflatoxins. It is based on the solubility properties of the toxin in the aqueous or organic phase or in their mixture. The disadvantage of this method is that it does not provide sufficiently clean analyte in all cases. Researchers have tested AFB1, AFB2, AFG1, AFG2, and AFM1 in breast milk with LLE, then high-pressure liquid chromatography (HPLC) with photochemical derivatization (PHRED) and fluorescence detection (FLD). The limits of the quantification (LOQ) were between 0.005 and 0.03 μg/kg (Andrale et al., 2013). Using the same procedure, LOQ of 0.01 μg/kg was obtained for AFB1 in rice and grain samples (Sheeijooni-Fumani et al., 2011; Biancardi et al., 2013) and co-workers got an LOQ of 15 ng/ml in skimmed milk matrix with HPLC/MS-MS measurement after LLE by using sodium chloride and ethyl acetate extraction agents. The average recovery of the method was 95% (*n* = 24; CV = 4,5%).

#### Liquid–Solid Extraction (LSE)

Liquid–solid extraction is a simple method for the extraction of aflatoxins from solid matrices of different consistency. The extraction steps include the weighing of homogenized sample of the appropriate particle size, adding the suitable extraction agent and then disintegrating the mixture applying, e.g., shaker, ultra-turrax, blender, vortex, or other methods to extract the components of interest. The extract, before analysis, is filtered and cleaned if necessary. An important step in the process is to select the most effective extraction solvent. The most commonly used extraction agents are mixtures of acetonitrile/water or methanol/water in different ratios (Sheibani and Ghaziaskar, 2009). For instance, the 80% methanol/water mixture proved to be the most optimal for extraction of aflatoxins in the case of nutmeg samples. The choice of methanol for further use (e.g., immunoaffinity chromatography, IAC) is also preferable, because the antibodies better tolerate higher concentrations of methanol than acetonitrile. Methanol was also suitable for chromatographic separation, as aflatoxins were measurable without interference (Kong et al., 2013). The efficiency of extraction is greatly influenced by the sample/solvent ratio, the composition of the extraction agent and the time of extraction. LSE alone is not satisfactory to extract aflatoxins without interference and further selective purification step(s) are usually required.

#### Ultrasound Extraction

The use of ultrasound can substantially increase the efficiency of LSE. Ultrasound extraction is most often implemented by immersing the vessel (e.g., Erlemeyer flask, centrifuge tube or vial) containing the sample to be extracted and the extraction solvent into an ultrasonic bath that contains water. During a few-minute treatment, the acoustic cavitation induced by the ultrasound significantly increases the transfer of the analytes and matrix components from the sample to the extraction solvent, thereby increasing the efficiency of extraction (Xie et al., 2016). According to Bacaloni et al. (2008) ultrasound treatment over 10 min did not significantly increase the efficiency of extraction in the case of hazelnut samples.

#### Pressurized Liquid Extraction (PLE)

The PLE procedure, also known as accelerated solvent extraction (ASE), is actually the same as LSE performed under increased pressure and temperature in a suitable pressure-resistant vessel. By selecting a vessel of appropriate size, samples of 1 to 100 g can be extracted. Naturally, in the case of test portions of a few grams, it is important to investigate the magnitude of the random and systematic errors resulting from the reduction of sample size, in order to avoid subsequent inadequate results. The advantages of the procedure are that the extraction process can be automated, and higher extraction efficiency can be achieved in shorter time and with lower amount of extraction solvent (Xie et al., 2016). This extraction method was successfully used in the case of aflatoxin analysis of pistachio samples (Sheibani and Ghaziaskar, 2009). This procedure increases the efficiency of extraction of the analytes from solid samples; nonetheless, it is not widely used because of the high price of the instrument.

### Supercritical Fluid Extraction (SFE)

Supercritical fluid extraction uses a supercritical CO_2_ fluid for the extraction of the required compound from the matrix. The SFE procedure is mainly used efficiently for the extraction of apolar organic molecules (Anklam et al., 1998). During the extraction of polar aflatoxins with SFE a number of problems have arisen, e.g., low recoveries and high concentrations of co-extracts. Furthermore, lipids may cause difficulties during further clean-up and chromatographic separation (Shephard, 2009). However, the SFE procedure was successfully used in the case of aflatoxin extraction from pepper (Ehlers et al., 2006) and from Ziziphy Fructus, a traditional Chinese medicine (Liau et al., 2007).

### Solid Phase Extraction (SPE)

Solid phase extraction is a popular clean-up method before qualitative and quantitative measurements of the components that have already been dissolved. Two types of SPE are used. In the case of the multi-step process (conditioning, sample application, washing, elution), either the measurand or the matrix component(s) is bound or removed from the sample (Yao et al., 2015). Various extenders are used in the SPE columns. Aflatoxins are often analyzed by using C-18 (octadecylsilane) column. The automated version of the procedure has been used for the online SPE ultra-high-performance liquid chromatograph coupled to a triple-quadrupole mass spectrometer (UHPLC-MS/MS) to determine aflatoxins from dried fruits. With this method, 83–103% recovery was achieved with RSD < 8, *n* = 3. These performance parameters are in line with EU requirements for determining mycotoxin levels in foods (Campone et al., 2018).

Special types of SPE procedures are **solid-phase micro extraction (SPME)** and IAC clean-up procedure that are based on the principle of immunoaffinity.

Compared to other extraction techniques, SPME has a number of benefits. Among others, it requires only sorption and desorption steps, it is a method easy to be automated, compatible with chromatographic systems, allows to achieve high enrichment, appropriate specificity can be assured, and it has very small sample requirements. The SPME method has been tested on the extraction of the aflatoxin content of nuts, spices, cereals and dried fruits. The result of the 8-min LC-MS measurement after clean-up with SPME method showed a sensitivity of 2.1–2.8 pg/ml for aflatoxins, which is more than 23 times greater than that achieved by the direct injection method (10 μl injection volume) (Nonaka et al., 2009). SPME was used for the clean-up of various types of cereal flours performed before the liquid chromatography and post-column PHRED-FLD measurements. The LOD and LOQ for aflatoxins were 0.035–0.2 ng/g and 0.1−0.63 ng/g, respectively (Quinto et al., 2009).

A specific application of SPE is the so-called immuno-affinity clean-up columns (IAC). They are applicable for the selective binding of mycotoxins as well. These columns contain selective antibodies produced against the mycotoxin to be analyzed and placed in the gel in the column. Chen et al. (2005) determined AFM1 in Pasteurized milk applying IAC cleanup and HPLC-FLD detection. In normal and low-fat content milks the average recovery and LOD were 78−79% and 0.59−0.66 ng/l, respectively.

**Multifunctional clean-up columns (MFC)** were designed for the simultaneous extraction of multiple types of mycotoxins (e.g., aflatoxins + zearalenone). The sample extract is pushed through the column and the lipophilic part of the packing binds fats and other non-polar matrix components, while the polar, ionic sites of the packing bind carbohydrates, proteins and other polar matrix components, while analytes pass through the column (Krska et al., 2008). There are dedicated columns commercially available for mycotoxin (aflatoxin) clean-up, e.g., MultiSep^®^, MycoSep^®^, and Myco6in1 column (Tang et al., 2013).

Others combined different IAC columns with hyphenated methods for selective clean-up of rye flour, maize and morning cereal samples (Wilcox et al., 2015). Immunoaffinity-based columns, applicable for multi-mycotoxin clean-up, were developed in recent years as a result of extensive research. Zhang et al. (2016) have developed IAC for AFB1, AFB2, AFG1, AFG2, Ochratoxin A (OTA), Zearalenone (ZEN) and T-2 toxins and tested agricultural products for them. By using acetonitrile/water/acetic acid (80:19:1, v/v/v) extraction, after multi-mycotoxin IAC, the samples were measured with HPLC-MS-MS. The linear ranges were 0.30−25, 0.12−20, 0.30−20, 0.12−20, 0.60−30, 0.30−25, and 1.2−40 μg/kg for AFB1, AFB2, AFG1, AFG2, OTA, ZEN and T-2, respectively. The LOD values were 0.1, 0.04, 0.1, 0.04, 0.2, 0.1, and 0.4 μg/kg, respectively. Hu et al. (2016) have developed immunoaffinity columns sensitive and specific for AFB1, AFB2, AFG1, AFG2, OTA, ZEN and sterigmatocystin T-2 toxins. This method allows the fast, simple and simultaneous determination of the above mentioned toxins in complex feed matrices after UPLC-MS-MS measurement. The LOD and LOQ of the method was 0.006−0.12 ng/ml 0.06−0.75 ng/ml, respectively.

Khayoon et al. (2010) used MFC columns successfully for the clean-up of aflatoxins from feed samples (Berthiller et al., 2017). This method is practical, portable and fast and requires no further clean-up steps (Wilson and Romer, 1991).

**Matrix solid phase dispersion (MSPD)** is a special type of SPE. It was developed as an alternative for the LLE procedure. Usually aluminum oxide, magnesium silicate or modified silica gel (C8, C18, amino, cyano) supports are used. It is particularly suitable for preparation, extraction and component fractionation of solid, semisolid and rather viscous biological samples (Cavaliere et al., 2007).

Matrix solid phase dispersion clean-up was used for aflatoxin analysis in olive oil samples with liquid chromatography electrospray ionization tandem mass spectrometric (LC/ESI-MS/MS) detection giving LOQ values between 0.04 and 0.12 μg/kg (Cavaliere et al., 2007).

The **Quick, Easy, Cheap, Effective, Rugged and Safe (QuEChERS)** method, developed for the extraction of pesticides with acetonitrile from vegetable samples, can be considered as a special alternative of the MSPD procedure (Anastassiades et al., 2003). Nowadays, with some modifications, it is widely used for mycotoxin clean-up as well (Xie et al., 2016).

Choochuay et al. (2018) developed a reliable and fast method for AFB1 determination in four feed types (broken rice, peanut, maize and fish feed). Sample preparation has been done by the QuEChERS method, then HPLC, precolumn derivatization and FLD were used. LOD was between 0.2 and 1.2 μg/kg and LOQ range was 0.3–1.5 μg/kg. The validated method was successfully used for the analysis of 120 samples The QuEChERS method has proved to be successful for the clean-up of AFM1, AFB2, AFG1, and AFG2 as well (Sartori et al., 2015).

#### Turbulent Flow Columns (TFC)

TurboFlow^TM^ technology is an automatic online sample preparation method for mass spectrometric analysis of complex matrices (Liang and Zhou, 2019). TurboFlow^TM^ technology combines the principles of diffusion, turbulence and chemistry in order to remove coextracted compounds from the matrix and capture the analyte rapidly and efficiently from the complex samples. It can be used with low input and high sensitivity in the case of difficult, multi-component samples. TurboFlow^TM^ columns have been tested for AFB1 and AFM1 in milk and milk powder samples. LOD was 0.05 μg/kg and LOQ was 0.1 μg/kg. Recovery of AFB1 and AFM1 was 81.1–102.1% for all samples (Fan et al., 2015).

#### Magnetic Nanoparticles Based Solid Phase Extraction (MSPE)

Magnetic nanoparticles based solid phase extraction based on the use of magnetic or magnetizable adsorbents can be used for the preconcentration of target analytes from large sample volumes (Safarikova and Safarik, 1999). Due to the diversity of the matrices to be tested, MSPE in itself is not sufficient for the extraction of aflatoxins from test samples, but in combination with other purification steps appropriate results can be achieved. Zhao et al. (2016) developed a two-step extraction technique combining ionic−liquid−based dispersive liquid–liquid microextraction and magnetic solid−phase extraction for the preconcentration and separation of aflatoxins in animal feedstuffs. After sample preparation HPLC-FLD was used for the detection of aflatoxins. Due to the rapid mass transfer associated with the steps of the dispersive liquid–liquid microextraction and the magnetic solid−phase extraction methods, fast extraction could be achieved. The detection limits (LOD) were 0.632, 0.087, 0.422, and 0.166 ng/ml for AB1, AFB2, AFG1, and AFG2, respectively.

## Separation Techniques

### Thin-Layer Chromatography (TLC), High-Performance Thin-Layer Chromatography (HPTLC)

At present, TLC is the best-known separation technique, but it may not be the most widely used anymore. Its popularity can be associated with its simplicity and low price, since its instrumental requirements at basic level are small. In preparative chemical laboratories TLC can be used to monitor the progress of reactions, determine the purity of a substance or identify compounds present in a given mixture.

In planar chromatography techniques, the stationary phase is an adsorbent material with different thicknesses through which the liquid mobile phase migrates via capillary forces. The most commonly used porous layers are silica gel, chemically modified silica gel, aluminum oxide (alumina), cellulose, chemically modified cellulose, polymer or ion-exchange resin. According to the phases we can differentiate between normal-, reversed- or mixed-phase plates.

HPTLC allows more selective and accurate quantitative measurements. The main differences between the techniques (TLC and HPTLC) can be derived from the differences in the particle size of the stationary phases, their sensitivity and data processing methods (Fuchs et al., 2010; Gurav and Medhe, 2018). When quantifying the concentration of aflatoxins on TLC plates coupled with fluorescent densitometry, the detection limit in red paprika, fish, maize and wheat was 0.5 μg/kg (Shephard, 2009). Corn samples spiked at 5 and 50 ng/g levels were measured by TLC separation and densitometric detection in an interlaboratory study. The relative repeatability standard deviation (RSDr) of the AB1 was between 56.6 and 41.7% (Park et al., 1994). Despite the fact, that TLC is still an accepted reference method for the detection of aflatoxins, the quantitative analysis of aflatoxins was replaced by HPLC and UPLC in most cases.

### Over-Pressured Layer Chromatography (OPLC)

Over-pressured layer chromatography was developed by Hungarian scientists in the mid-70s (Tyihák et al., 1979; Kalász et al., 1980; Tyihák et al., 1981; Hauck and Jost, 1983).

Over-pressured layer chromatography is carried out on a TLC or HPTLC plate, applying forced flow in a pressurized ultramicro (UM) chamber, based on the principle of liquid chromatography (Tyihák and Mincsovics, 2011).

Over-pressured layer chromatography integrates the advantages of classical TLC and HPLC, namely the possibility of parallel analysis in thin layer chromatography and the application of forced flow used in HPLC (Tyihák et al., 1979).

The applicability of OPLC for aflatoxins was proven in a validation procedure carried out by the scientists who developed the technology. As a result, the following LODs were defined for aflatoxins: 0.018, 0.100, 0.15, and 0.14 μg/kg for AFG2, AFG1, AFB2 and AFB1, respectively (Papp et al., 2000).

### High/Ultrahigh Performance Liquid Chromatography (HPLC/UHPLC)

The reference methods for the detection of aflatoxins are based on chromatography, more precisely on HPLC/UPLC. During the determination of aflatoxins HPLC-fluorescent detection (FLD) and HPLC-MS/MS systems can be used in most cases. If the separated components are detected with fluorescent detector, there is a need for post-column derivatization (PCD) in order to increase the natural fluorescence properties of AFB1 and AFG1. This derivatization can be based on electrochemical or photochemical principles. For electrochemical derivatization trifluoroacetic acid (TFA), potassium bromide (KBr) or iodine can be used as reagent.

After MultiSep # 228 column clean-up Akiyama et al. (2001) applied TFA derivatization with LC FLD in red pepper for aflatoxin detection. With this derivatization technique 0.5 μg/kg LOD was measured for red pepper.

Post-column derivatization (PCD) including electrochemical bromination is considered as a widely used method for the analysis of aflatoxins. PCD can be achieved with either pyridinyl hydrobromide perbromide (PBPB) or with an electrochemical cell (KobraCell) where KBr is added to the mobile phase. Both derivatization techniques were used in several laboratories to analyze baby foods. When evaluating the results, no significant differences were found between the two PCD techniques. The recoveries ranged from 92 to 101%. During the laboratory analyses the technique resulted in an LOD of 0.02 μg/kg, LOQ of 0.1 μg/kg for AFB1 in baby food (infant formula) samples (Stroka et al., 2001; Gilbert and Vargas, 2003).

For enhancing the fluorescence properties/response of aflatoxins, PCD using iodine can also be considered as a method for aflatoxin detection. A great disadvantage of PCD using iodine is that the derivatization capability of iodine constantly reduces over time and, consequently, there is a parallel decrease in the sensitivity of the technique. The method yielded reproducible results at 1 μg/kg LOD for peanut butter samples.

Aggressive chemicals (e.g., KBr), however, which shorten the lifespan of instruments and capillaries, can be replaced by PHRED. Significant features of detection of aflatoxins with PHRED and FLD are 0.004 μg/kg (LOD) and 0.015 μg/kg (LOQ) (Rahmani et al., 2013). HPLC with FLD and in-line photochemical reactor is capable of determining aflatoxins separately in low μg/kg concentrations. An advantage of the method is that reagents for the sensitive measurement and substances for derivatization are not needed. The latter is based on the fact, that upon irradiation by 254 nm ultraviolet (UV) light, fluorescent properties of AFB1 and AFG1 components are increasing equivalently to electrochemical derivatization (Papadopoulou-Bouraoui et al., 2002).

There are further possibilities for the fluorescence-based detection of aflatoxins, e.g., HPLC-LIF. Laser-induced fluorescence (LIF) is based on the analysis of fluorescent light emitted during laser irradiation. Sensitivity of the method is 0.1 μg/kg for AFB1 and AFG1, and 1.2 μg/kg for AFB2 and AFG2 (Gan et al., 1989; Gilbert and Vargas, 2003). Its application is not widespread as HPLC-FLD is a cheaper and suitable technique for the detection of aflatoxins. UV detection is often mentioned in the literature besides fluorescence, but this procedure is not widespread in routine analysis. HPLC-UV determination was performed in egg and liver matrices, where the LOD and LOQ for AFB1 were 0.08 and 0.28 μg/kg (Amirkhizi et al., 2015). Aflatoxins can be detected by UV absorption; however, it is not sufficiently sensitive in all cases to reach the μg/kg range. Spectrometric detection will be discussed later.

Derivatization is not needed for the analysis of AFM1 occurring in milk and dairy products, as this component can be analyzed with HPLC-FLD with sufficient sensitivity. AFM1 determination was performed in milk and milk powder samples by using OASIS^TM^ Hydrophilic-Lipophilic Balance (HLB) SPE clean-up column, C-18 reversed-phase HPLC column and FLD detection, which is a simple and not the most expensive method. The detection limit/quantification limit of this method was 0.006/0,026 μg/kg for milk and 0.026/0.087 μg/kg for milk powder (Wang et al., 2012). The recovery was 85.4−96.9%. AFM1 was analyzed in milk, yogurt and cheese matrices with IAC clean-up, reversed phase HPLC separation and FLD detection, where the limit of determination for AFM1 was 0.003 μg/kg in milk, 0.07 μg/kg in yogurt and 0.05 μg/kg in cheese. The recovery was 85.4−96.9% (Yoon et al., 2016).

### Electric Driven Techniques

Capillary electrophoresis (CE) is in fact a range of separation techniques based on different separation principles: capillary zone electrophoresis – CZE (based on differences between electrophoretic mobilities of analyses), micellar electro-kinetic capillary chromatography – MEKC (partition of neutral compounds with surface active micelles), capillary gel electrophoresis – CGE (filtration of analytes through a gel network), capillary isoelectric focusing – CIEF (separation of zwitterionic analytes with pH gradient), capillary electrochromatography – CEC (separation of compounds on a column packed with silica gel particles using electric field) (Hancu et al., 2013).

The classic CZE method, which is based on the differences between the electrophoretic mobilities of the analytes, is unfit for the separation of neutral compounds, which migrate with the same rate as the electro-osmotic flow (EOF) (Hancu et al., 2013).

Based on a hybrid method combining chromatographic and electrophoretic separation principles, micellar electro-kinetic capillary chromatography (MEKC) extends the applicability of capillary electrophoretic methods to neutral analytes. In the case of MEKC, surface-active compounds are added to the buffer solution in a concentration exceeding their critical micellar concentration. Consequently, they form micelles, which affect the electrophoretic migration, like any other charged particle. The separation is based on the differential distribution of the analyte between the two phases of the system: the mobile liquid phase and the micellar pseudostatic phase (Hancu et al., 2013). Aflatoxins were measured with the MEKC procedure in the feed of milking cows, including alfalfa, wheat bran and maize grains. Aflatoxins were separated in a silica capillary, and fluorescence was induced by 355 nm UV light. LODs and LOQs were between 0.002–0.075 and 0.007–0.300 μg/kg for the four aflatoxins, with analysis time within 6.5 min. The recovery was 70−108% (Gao et al., 2019). Six mycotoxins were determined with high reproducibility from feed samples, with the use of the MEKC procedure. The LOD/LOQ values were between 0.02/0.12 and 0.06/0.42 μg/kg, the recovery was 80−130% (Peña et al., 2002). Modified methods of MEKC, among others, are reversed-flow micellar electrokinetic chromatography (RFMEKC) and capillary electrokinetic chromatography (CEKC) with multiphoton excited fluorescence (MPE) detection (Gilbert and Vargas, 2003). CEC or CEKC are procedures to be applied for the separation of big molecules; however, no validated method was found. CE and, in particular, MEKC with laser-induced fluorescence detection (MEKC-LIF) appeared to be interesting techniques for determination of aflatoxins for a while, but no applications can be found in routine analysis (Naushad and Khan, 2014). The techniques mentioned above can be coupled with other detection systems, such as MEKC-fiber-optic sensor (SBFOS) (Dickens and Sepaniak, 2000).

### Hyphenated Techniques

Hyphenated techniques usually mean separation procedures connected to a mass spectrometer. Of these, LC/UPLC-MS, SFC-MS, CE-MS and Chip-MS techniques have been used to determine aflatoxins. These procedures are presented below.

#### Liquid Chromatography/Ultra-Performance Liquid Chromatography Mass Spectrometry (LC/UPLC-MS) and Tandem Mass Spectrometry (MS/MS)

Until the early 1990s, thermospray, particle beam and fast atom bombardment interfaces were used for the LC/MS measurement of mycotoxins (Zöllner and Mayer-Helm, 2006). Using these interfaces, however, sensitivity and ionization efficiency problems often occurred. A breakthrough came in the beginning of 1990s, when the first instruments equipped with atmospheric pressure ionization sources (API) appeared on the analytical market. For the past 3 decades, both LC/UPLC-MS and MS/MS systems have become basic apparatus in almost all well-equipped research and routine laboratories of organic analytics. Due to their versatile applicability, these instruments are increasingly used in mycotoxin analytics as the sole qualitative/quantitative methods or as confirmatory methods to accurately determine the mycotoxin content of samples found to be positive at the screening by rapid methods (such as ELISA, Lateral Flow).

It needs to be mentioned, however, that the wider proliferation of these methods is hindered by their high price and the costs of training personnel for their professional operation and method development.

#### Atmospheric Pressure Ion Sources for the Determination of Aflatoxins by LC/UPLC-MS and MS/MS

LC-MS analysis of aflatoxins is possible with the application of all three commonly used atmospheric pressure ion sources. Review publications reveal that the atmospheric pressure electrospray (ESI) source is used predominantly for the LC-MS determination of aflatoxins (Zöllner and Mayer-Helm, 2006; Li et al., 2013; Yao et al., 2015). One reason for this is that ESI ionization of aflatoxins is very effective and the protonated molecules ([M + H]^+^) and fragment ions created in the collision zone (CID) in the case of MS/MS can be measured well. Another reason is that users usually don’t purchase the atmospheric pressure photoionization source (APPI) for most LC-MS instruments, or in the case of purchase, they don’t have sufficient experience with its application. Atmospheric pressure chemical ionization (APCI) has also been successfully used for the sensitive LC-MS determination of aflatoxins (Abbas et al., 2002, 2006; Pacheco and Scussel, 2007; Xie et al., 2016).

If only aflatoxins need to be determined in samples to be tested, APPI can be considered to be the best choice among atmospheric pressure ion sources, as it has considerably lower background noise and ion suppression compared to ESI and APCI. The reason is that in the case of direct photoionization (direct APPI), only components with ionization potential (IP) value below the energy of photons emitted by the vacuum UV lamp of the ion source (10 eV) are ionized in the ion source. In other words, significant portion of matrix components and potential contaminants in the mobile phase will not give noise during photoionization (signal enhancement/ion suppression). It was found that a mass spectrometer will be 2–3 times more sensitive during aflatoxin measurement, if equipped with APPI instead of ESI ion source (Takino et al., 2004; Cavaliere et al., 2006). It must also be noted, however, that the so called multitoxin methods based on LC-MS/MS are spreading increasingly (Berthiller et al., 2007; Li et al., 2013; Xie et al., 2016; Zhang et al., 2016; Malachová et al., 2018). These methods need to use ESI ion source, being the most effective to measure all mycotoxins, which are officially regulated. Furthermore, most mycotoxins will not give sufficient signal when detected by MS or MS/MS with APPI ion source.

#### Mass Analyzer Types for the LC/UPLC-MS and MS/MS Determination of Aflatoxins

**FIGURE 2:**
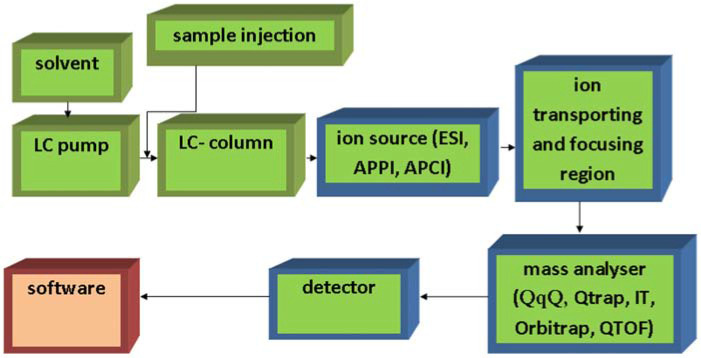
Simple schematic of LC-MS system.

Leaving the atmospheric pressure ion source, the ionized molecules enter the vacuum chamber of the mass spectrometer, and they reach the actual mass filter/mass analyzer through an iontransporting and focusing region. The mass analyzer can be single-stage or multi-stage (MS/MS) (Figure 2).

Due to the lack of collision-induced dissociation (CID), the fragmentation of molecular ions is not possible in mass spectrometers equipped with single-stage mass analyzers (e.g., single quadrupole) (with the exception of in-source CID), which would be prerequisite to the MS/MS spectrum based identification and exact determination of components eluting from the LC/UPLC column. Single-stage type mass analyzers are not compliant with EU requirements of residue analysis, requiring a precursor ion, two product ions and their ratio for the MS identification of a component (EU, 2002). Mass spectrometers equipped with multi-stage mass analyzer are compliant with these conditions. Several mass spectrometers equipped with multi-stage mass analyzer (MS/MS) have been applied for the analysis of aflatoxins: triple quadrupole (QqQ), 3D ion trap, quadrupole-linear ion trap (Q-TRAP), quadrupole-time of flight (Q-TOF), and orbitrap. Moreover, the availability of instruments equipped with these mass analyzers allowed the development of multitoxin procedures mentioned previously.

The most widespread and one of the best solutions for the quantitative determination of organic compounds with hyphenated techniques (e.g., LC/UPLC-MS/MS) is certainly the application of mass spectrometers equipped with triple quadrupole (QqQ) mass analyzer. LC/UPLC-QqQ-MS procedures are the most widespread among multitoxin methods (including aflatoxins, too) (Zöllner and Mayer-Helm, 2006; Herebian et al., 2009; Wei et al., 2013; Zhang et al., 2016; Malachová et al., 2018). Zhang et al. (2016) investigated the occurrence of 7 mycotoxins (including AFB1, AFB2, AFG1 and AFG2) in peanut, maize and wheat samples after IAC clean-up using the multitoxin LC-ESI-QqQ-MS/MS procedure. The LOD/LOQ values of the four mycotoxins were 0.1, 0.04, 0.1, 0.04/0.3, 0.12, 0.3, and 0.12 μg/kg. The recoveries were between 95.3 and 103.3%. Huang et al. (2014) investigated milk samples (row milk, liquid milk, milk powder) with UPLC-ESI-QqQ-MS/MS multitoxin (including aflatoxin) method after SPE. LOD values were 0.001–0.003 μg/kg, while LOQ values were between 0.003 and 0.015 μg/kg with recoveries ranged between 87 and 109%. Wei et al. (2013) elaborated a procedure with IAC clean-up followed by LC-ESI-QqQ-MS/MS for aflatoxin and ochratoxin A analysis in licorice (*Glycyrrhiza uralensis*) samples. For AFB1, AFB2, AFG1, AFG2 the LODs were 0.007, 0.005, 0.003, 0.005 μg/kg; while the LOQs were 0.020, 0.015, 0.010, 0.015 μg/kg, respectively. The recoveries ranges between 72.7 and 123.3%. McCullum et al. (2014) investigated the aflatoxin contamination of red wine samples with MSPE followed by the LC-ESI-QqQ-MS/MS method. The calibration curve was linear in the 0.006–3 ng/ml range. LOD values for AFB1, AFB2 and AFG1 toxins were 0.0012 ng/ml and 0.0031 ng/ml for AFG2. Mass spectrometers equipped with QqQ mass analyzer have excellent sensitivity and selectivity, but in quantitative measurement, usually the third quadrupole is also working in selected ion monitoring (SIM) mode; therefore, the information needed for structural identification is lost (Hernández et al., 2005).

If necessary, this information can be acquired by the application of a hybrid mass spectrometer such as a quadrupole-linear ion trap (QTRAP^®^) equipment, which enables both quantitative determination and confirmation based on the mass spectrum (Martínez Bueno et al., 2007).

LC-MS/MS having QTRAP^®^ mass analyzer has been applied for multi-toxin measurement of aflatoxins in baby food. LOD and LOQ values ranged between 0.05–0.4 and 0.1–1 μg/l for the four aflatoxins (AFB1, AFB2, AFG1, and AFG2); the recovery was 78% (Rubert et al., 2012). This mass analyzer together with APPI ionization source has also been used for the detection of AFM1 toxin in very low concentrations in milk without observing any significant matrix effect. LOQ values ranged between 0.006–0.035 μg/l; note, however, that LOD values were not reported.

For aflatoxin analysis, LC-MS instruments including the so-called 3D iontrap (IT) mass analyzer have already been used. Cavaliere et al. (2006) determined AFM1 in milk samples. The LOD and LOQ were 1 and 6 ng/kg compared to 3 and 12 ng/kg obtained with ESI ion source. The recovery was between 92 and 98%.

Lattanzio et al. (2007) investigated 11 mycotoxins, including aflatoxins (AFB1, AFB2, AFG1, and AFG2) from maize extracts with multitoxin immunoaffinity sample clean-up followed by LC-ESI-IT-MS/MS procedure. LOD values of 0.3–4.2 μg/kg were found for mycotoxins with average recovery of 79%. Schatzki and Haddon (2002) applied an IT-MS device without clean-up for the screening of aflatoxin content of 65,000 walnut samples. Aflatoxin contamination was found in 120 samples in the concentration range of 250–43,000 ng/g.

Saldan et al. (2018) coupled a quadrupole–time-of-flight (QTOF) mass spectrometer to a liquid chromatograph (LC-QTOF-MS) for the identification of *Aspergillus flavus* strains grown on agar medium, based on chemical markers (secondary metabolites including AFB1, AFG2). LOD and LOQ values ranged between 0.1–0.3 μg/kg and 0.2–0.9 μg/kg for the identified components during the analysis of the culture extracts.

Herebian et al. (2009) combined micro-LC separation with a mass spectrometer containing a linear trap quadrupole (LTQ)-Orbitrap mass analyzer for multitoxin determination, where the AFB1, AFB2, AFG1, and AFG2 contents of wheat and maize extracts were also analyzed. The measurement was performed in full scan mode by determining the accurate mass of extracted ions. LOD for mycotoxins was between 0.4 and 2000 ng/ml. Specific LOD values for aflatoxins, however, were not reported.

The ion suppression/enhancement caused by the matrix effect can rarely be avoided even by these sophisticated multi-stage mass analyzers, particularly, when the raw sample extract is analyzed by LC/UPLC-MS/MS without clean-up (“extract and shoot” method). To avoid such problems and reduce the LOD/LOQ values, the sample clean-up procedures discussed above are extensively used before the LC/UPLC-MS/MS measurement of mycotoxins, including aflatoxins. Prominent procedures of these are the IAC clean-up (Dragacci et al., 2001; Mazaheri, 2009; Xie et al., 2016; Zhang et al., 2016) and QuEChERS (Anastassiades et al., 2003; Xie et al., 2016) discussed above. It also needs to be mentioned, that to increase the accuracy of quantitative evaluation, at least the so-called external matrix-matched calibration needs to be performed. However, the best solution used currently is to add isotope-labeled internal standards of the mycotoxins by an automatic sample injector to both the matrix-matched calibration samples and samples to be measured (Zöllner and Mayer-Helm, 2006). Obviously, the application of isotope-labeled internal standards, particularly for multitoxin analysis, results in significant cost increase (Li et al., 2013; Šarkanj et al., 2018).

#### Supercritical Fluid Chromatography Mass Spectrometry (SFC-MS)

The SFC technique combines the numerous advantages of liquid and gas chromatography. Its application is beneficial for non-volatile, heat sensitive, reactive and multicomponent samples. SFC provides results faster than HPLC, because diffusion of the substance is 10 times faster in the supercritical solvent (CO_2_) than in liquid phase. The analysis is usually performed in environmentally benign manner without the use of organic solvents; however, MeOH or a 1:2 MeOH:ACN mixture is added to CO_2_ as a polar modifier if necessary (Taylor et al., 1997). The separation process takes place at a lower temperature than in the case of GC, and with similar efficiency. Its disadvantage is its very high price; therefore, SFC procedures have been developed for the determination of relatively few compounds.

The SFC procedure combined with a tandem mass spectrometer containing ESI ion source (SFC-MS/MS) has been used for the simple, fast and sensitive determination of aflatoxins in edible oil (Lei et al., 2016). CO_2_–methanol gradient elution was used to the baseline separation of the four aflatoxins. Following separation, there was a need to use post-column make-up flow before the introduction into the ESI ion source, to achieve a sensitive SFC-MS/MS determination of the components. The LOD and LOQ values for aflatoxins ranged in order 0.02−0,04 and 0.05–0.12 μg/l, while RSD was lower than 8.5%. Applying internal standard a recovery of 98% was achieved.

#### Chip-MS

In the first chip-MS-based system for AFB1 determination, a plastic microfluidic chip was used for the automatic affinity dialysis, concentration and subsequent ESI-MS determination of reaction mixtures containing AFB1 antibodies and aflatoxins (Yiang et al., 2001).

For the determination of aflatoxins in peanut products, a procedure was also developed, where a nano LC pump was coupled to a QqQ-MS through a chip-ESI-MS ion source (chip-nano LC) (Liu et al., 2013). Following solvent extraction, immunoaffinity solid-phase clean-up was carried out to reduce the matrix effect. Separation was performed by gradient elution and detection was done using multiple reaction monitoring. Linear dynamic range for the four main aflatoxins was 0.048–16 ng/g. LOD was reported to be between 0.004 and 0.008 ng/g. Accuracy (96.1%-105.7%/95.5%-104.9%) were obtained.

Beside the sensitivity of determination and the low amounts of sample needed, the significance of the chip-MS procedure is its environmentally benign manner resulting from low solvent consumption. Due to decreasing prices of the chips and instruments, the spreading of these methods is to be expected.

### Rapid Test Methods

Rapid tests developed for the analysis of aflatoxins are built upon several different technologies. The most common ones are the enzyme-linked immunosorbent assay (ELISA), lateral flow devices (LFD) and chemical methods. Rapid tests are indispensable to provide analytical results within a short time. These procedures enable the analysis to be easily performed with lower prices, even at the location of sampling.

The vast majority of the rapid methods used for aflatoxin measurement are immunoassays based on the reaction of a special antibody and the antigen of the analyte, which can be detected by various markers.

#### Markers

Many markers have been developed over the years, including enzymes, radioisotopes, fluorophores, gold nanoparticles and other sensitive optical and electrochemical components (Mataboro et al., 2017).

##### Enzyme label Enzyme-linked immunosorbent assay (ELISA)

**FIGURE 3:**
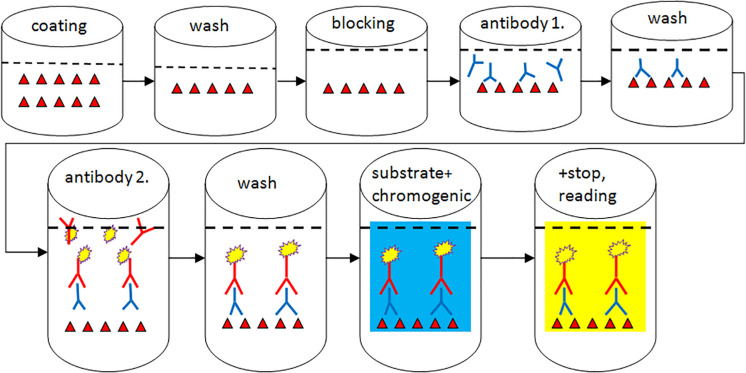
Structure of a competitive ELISA.

The aim of the ELISA technique is the qualitative or quantitative determination of mycotoxins found in the analytical sample, based on the application of antibodies, which are specific to compounds to be analyzed. The method is based on an enzyme-linked color reaction. For the detection of mycotoxins, competitive-type ELISA tests are typically used. Consequently, the measured color intensity is inversely proportional to the concentration of the measured compound (Waliyar et al., 2009; Figure 3).

These ELISA analytical systems are excellent screening devices, provide quantitative results in a short time period, and as previously mentioned, they can often be used at the location of sampling, too. However, cross-reactions with molecules very similar to the analyzed substance and matrix effects found during the analysis of different products may influence the results. Naturally, quantitative determination of AFB1, AFT and AFM1 can also be performed with the ELISA technique (Ketney et al., 2017). The producers of the tests have considered the different regulatory limits of different regions. A substantial part of agricultural raw materials can be analyzed with the ELISA technique, according to the guidance provided by the producer, without the application of particular cleaning steps. ELISA analysis of more complicated sample types, like compound feed, however, may provide inaccurate results. In order to avoid this situation, it is recommended to consult the producer of the tests concerning the sample to be analyzed. Alternatively, the process is recommended to be individually validated for the matrices to be tested. However, if the measurement of a complex matrix is needed, which is not on the list of substances validated by the producers, or if the aim is to confirm the result of a rapid test, the sample has to be analyzed with reference methods (Andreasson et al., 2015).

The sensitivity of the ELISA kit depends on the manufacturer. For instance Romer Labs Inc. United States reported an LOD of 0.018 μg/kg and LOQ of 0.025 μg/kg with recoveries ranging between 80 and 120% for the determination of AFM1 in milk.

An improved version of ELISA is **(Tumor Specific Antigen) TSA-ELISA**, where the intensity of the sign generated by ELISA can be increased several folds by the addition of tyramide. Under optimal circumstances, the LOD, IC10 and the half maximum inhibition concentration (IC) (IC50) of TSA-ELISA is 0.004 and 0.039 ng/ml, respectively, in the case of AFB1. The elaborated TSA-ELISA method afforded LOD values 11 times better and IC50 values 6 times better compared to those measured by the traditional ELISA method in the analysis of AFB1 in edible oil samples (Zhang et al., 2018). TSA-ELISA is a satisfactory, sensitive and cheap method with good reproducibility, and a useful alternative for AFB1 detection in edible oil samples.

##### Radioimmunoassay (RIA)

Radioimmunoassay applies radioactively labeled molecules during the stepwise formation of immunocomplexes. RIA is a highly specific and very sensitive method. In the case of agricultural samples (maize, soybean, wheat and rice), the LOD/LOQ of the method was 0.2/0.5 μg/kg for AFB1. The recovery was between 92 and 107% (Korde et al., 2003).

RIA requires the application of an expensive, special equipment to minimize the adverse effects caused by gamma rays (Waliyar et al., 2009).

For this reason, in order to avoid health risks, other types of marker compounds might be more beneficial for the analysis of aflatoxins (Hemmilä, 1985).

##### Fluoroimmunoassays (FIA)

Immuno reagents with probes based on fluorescent labeling are already used widely. By combining the highly sensitive fluorescence method with the sensitivity of the measuring instrument, a simple and rapid analytical procedure can be achieved, where the concentration of the analyte can be directly measured in the reaction mixture. The problem with FIA methods was the low sensitivity caused largely by the high background noise of the fluorometric measurement (Hemmilä, 1985). The background has been reduced by continuous improvements, e.g., solid-phase separation systems, new fluorescent probes and new instruments **time-resolved fluoroimmunoassays (TRFIA)**, resulting in a sensitivity, which is suitable to analyze mycotoxins today. It was demonstrated that under optimal analytical conditions, TRFIA was very sensitive and specific to detect AFB1 with an LOD of 0.1 μg/kg in feed samples. TRFIA demonstrated high accuracy during the determination of AFB1 in feed samples. Average recovery ranged between 93.71% and 97.80% with a coefficient of variation of 1.25–3.73%. A very good correlation was found between TRFIA and HPLC methods during AFB1 determination of feeds, which confirmed the reliability of the developed method (Hu et al., 2018). Wang et al. (2016) determined AFB1 toxin from soy sauce with TRFIA technique. The range of the measurement was between 0.3 and 10.0 μg/kg, the LOD value was 0.1 μg/kg. The recovery was between 87 and 113%.

**Flow cytometry based competitive fluorescent microsphere immunoassay (CFIA)** is a microbead-based competitive fluorescent immunoassay applying monoclonal antibodies of high affinity. It can simultaneously detect six mycotoxins (OTA, AFB1, FB1, DON, T2, ZEA) with increased sensitivity for aflatoxins (0.12 μg/kg) following a simple extraction procedure compared to an ELISA method (Czéh et al., 2012; Czéh, 2014; Bánáti et al., 2017).

##### Chemiluminescence immunoassay (CLIA)

Chemiluminescence immunoassay is an immunoanalytical technique, where the marker is a luminescent molecule. Luminescence is usually the emission of visible or near visible (λ = 300–800 nm) radiation. The advantage of luminescence in spectrophotometry over absorption is that its signal is absolute, while the latter one is relative. Chemiluminescence methods can be direct, by using luminophores as markers or indirect, by using enzyme markers. Each of them can be competitive or non-competitive. Fang et al. (2011) developed a CLIA technique for the analysis of AFB1 in agricultural products. The method had a LOD of 0.01 ng/g and a linear range of 0.05 to 10 ng/g with 79.8−115.4% recovery.

##### Other

In some areas of analytics, color label markers (e.g., gold nanoparticles, colored latex) are the most widely used for rapid and qualitative determination. In addition to the above mentioned markers, aflatoxins can also be made fluorescent by irradiation with UV or laser light. However, they may also be derivatized with various chemical agents (e.g., iodine, bromine, etc.) (Li et al., 2009).

#### Immunological Devices

The most widely used immunological devices are microplate-based immunoassays, lateral flow immunoassays (LFIAs) and different biosensors (immunosensors) (Li et al., 2009).

##### Microplate−based immunoassays

When analyzing aflatoxins, microtiter plate and reader-based immunoassays allow simultaneous analysis of many samples, since the plates used have multiple wells. Most widely used microplate-based immunoassays are ELISA, fluorescence and chemiluminescence based analyses (Li et al., 2009).

##### Lateral flow immunoassay (LFIA), (LFA), or lateral flow devices (LFD)

Immunochromatographic dipsticks are another appropriate technology on the market of rapid mycotoxin tests. The basis of the method is the detection of the analyzed component by linking to a specific antibody in the test zone, which is placed on a membrane fixed on the dipstick. In addition to the test zone there is the control zone on the membrane verifying the correct functioning of the test. When the sample extract flows on the membrane, it passes the test and control zones and, depending on the concentration of the toxin, both (test and control) lines or only the control line will become visible. The dipstick can be evaluated visually by the naked eye or with the help of a reading device. When quantitative results are needed, the evaluation is performed by an instrument (reflectance photometer), which measures the intensity of the test and control lines and evaluates the results on the basis of data determined. The immunochromatographic dipstick is a rapid, easy-to-perform technique, which is ideal and cost-effective even for the analysis of a single sample. Similar to the ELISA technique, cross-reactions and matrix effects occurring during the analysis of certain products limit the applicability of the dipstick. For the determination of aflatoxins, qualitative, and quantitative immunochromatographic dipsticks are available (Anfossi et al., 2013). These tests have basically been validated for simple sample matrices; thus, their application is recommended for the screening analyses of raw materials.

**FIGURE 4:**
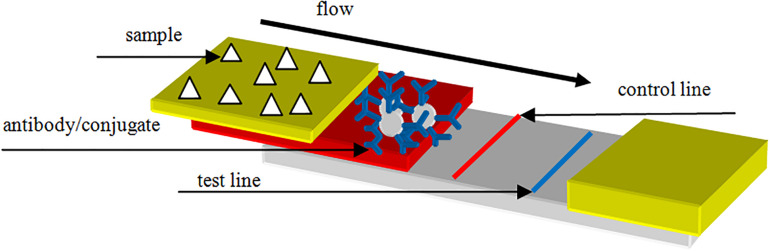
Structure of lateral flow immunoassay.

**FIGURE 5:**
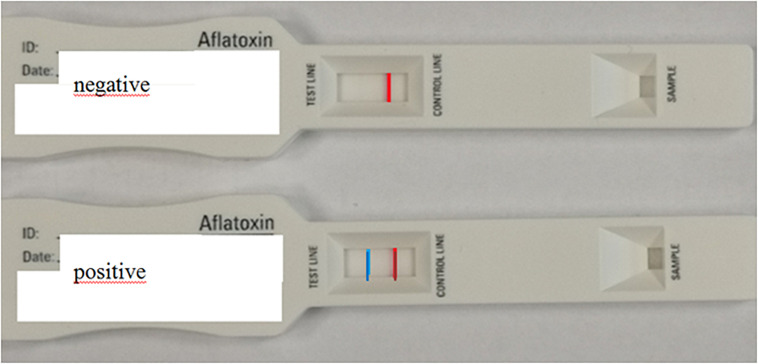
Practical application of the lateral flow immunoassay.

However, results are available from the analysis of certain more complex matrices as well. The visual detection limit for AFB1 in this case was 5 μg/kg (Delmull et al., 2005). A decision level of 0.1 μg/kg was achieved with LFIA technique in food samples (Liao and Li, 2010; Figures 4, 5).

The detection options of LFIA depends on the type of the marker. In case of color label markers (e.g., gold nanoparticles, colored latex), besides instrumental reader, there is a possibility of visual evaluation, while in case of fluorescence (e.g., quantum dots, ruthenium complexes) or other markers (e.g., enzyme labels or paramagnetic labels), only readers or expensive detectors can be used for quantification (Koczula and Gallotta, 2016).

##### Chromatographic time-resolved fluoroimmunoassay (CTRFIA)

A portable immunosensor based on chromatographic time-resolved fluoroimmunoassay has been developed for fast on-site sensitive determination of AFB1 in food and feed samples. CTRFIA provides an increased positive signal and low signal-to-noise ratio in time-resolved mode. Zhang et al. (2015) applied the method to various food and feed matrices such as maize (LOD 0.06 μg/kg), peanut (LOD 0.09 μg/kg) and vegetable oil (LOD 0.09 μg/kg). These matrices yielded a recovery of 116.7% from 80.5%. Tang et al. (2015) showed a fast method without sample preparation that can be performed just within 6 minutes. Its LOD for raw milk AFM1 matrix was 0.03 ng/ml, the measurement range was between 0.1 and 0.2 ng/ml and the recovery in case of quantitative determination was 80−110%. Tang et al. (2017) also measured simultaneously AFB1 and ZEN in maize with CTRFIA method. The LOD values of the method were 0.05 ng/ml for AFB1 and 0.07 ng/ml for ZEN.

LFIA is considered as a fast and sufficiently sensitive screening method. The need for the development of multi mycotoxin analysis has arisen in this research area as well, as this method was previously only applicable for one mycotoxin analysis at a time. The publication of Zhang et al. (2018) describes a multicolor-based immunochromatographic strip (ICS) semi-quantification method that is suitable for the simultaneous determination of 3 mycotoxins (AFB1, ZEN, T-2). Maize and cereal-based feed matrices were analyzed. Visual LOD-s estimated by the researchers were 0.5, 2, and 30 ng/ml for the above-mentioned toxins, respectively. The cut-off values were 1, 10, and 50 ng/ml respectively.

##### Biosensors

Chemical sensors are small-size devices, which convert the chemical information characterizing the composition of the compound into electronic or optical signal by continuous tracking, in real time. Such sensors represent modern analytical devices of our days. They take over the role of traditional analytical methods in several areas, since they can be well miniaturized due to their robust structure, can be integrated in automatic systems, and can be applied in *in situ* analysis as well. Chemical sensors usually lag behind laboratory instruments regarding analytical performance parameters of selectivity, sensitivity and stability. For this reason, the requirements of the area of application should be borne in mind during the development of sensors. Their grouping is usually based on the functioning of the transducer system or on the substance to be measured (e.g., gas-, ionic-, biosensor).

###### Label based biosensors

Biosensors, a sub-group of chemical sensors, are special selective analytical devices, which are closely linked to or integrated into a physico-chemical transducer (e.g., electrochemical, optical, piezoelectric, etc.) and contain a substance of biological origin (e.g., enzyme, tissue, microorganism, antibody, etc.) or an imitating substance (e.g., molecularly imprinted polymers, MIP) (Sharma et al., 2003).

Detection is based on the linking of the analyte to its specific complementary biological element (bioreceptor), which is fixed on a suitable portable surface (Velasco-Garcia and Mottram, 2003). The rise of biosensor techniques can be explained by their many advantages compared to conventional analytical techniques. The selectivity provided by the biologically based element grounds the development of specific devices, which often facilitate real-time analyses of small amounts of complex samples, with simple sample preparation. The MIP procedure was successfully used for the selective extraction and pre-concentration of AFB1 in infant food sample. The LOD was 0.0275 μg/kg with recoveries of 83.51−90.03% (Semong and Batlokwa, 2017). The sensor developed by Jiang et al. (2015) showed a wide linear range between 1 fg/ml and 1 μg/ml. In the rice sample the LOD of AFB1 was 0.3 fg/ml and the LOQ was 1 fg/ml. Depending on the type of label, highly sensitive and selective analyses include, among others, FIA, RIA and EIA. See Section “Rapid Test Methods.”

###### Label-free biosensors

Techniques based on labeling molecules are increasingly lagging behind in the area of measurement of interactions between different molecules in biological and biochemical systems. Surface plasmon resonance (SPR) is a distinguished method among label-free analytical methods, which can analyze the interactions near surfaces, based on the SPR phenomenon. It can indicate not only the endpoint, but the whole process can be monitored.

Mass-change-based sensors most often use mechano-acoustic sensors based on the change of resonance frequency, with label-free techniques of quartz crystal microbalance (QCM) and optical waveguide light-mode spectroscopy (OWLS). Similar to other label-free detection methods, OWLS enables the real-time inspection of molecular-level processes at the interface. This can be achieved by the application of the two-part integrated optical waveguide sensor (chip), which is the basis of the technique. A sensitive method could be developed for mycotoxins including aflatoxins from pepper, applying gold nanoparticles of different sizes and origin (Adányi et al., 2018). When analyzing aflatoxins with OWLS, the LOQ for AFB1 in wheat, barley and pepper samples was between 0.001–1 μg/kg, while the LOD was 0.0005 μg/kg with 76.4−108.6% recoveries (Adányi, 2013). Its disadvantage is that although it is sensitive, it is not selective in the case of complex samples. However, the required selectivity can be achieved by prior sample clean-up with immunoaffinity column, providing a clean solution without interferences (Majzik et al., 2015).

###### Lab-on-a-chip based biosensor (LOC)

Lab-on-a-chip is a device, which integrates one or more laboratory functions into one chip, having a size of only a few square centimeters. LOCs are able to manage extraordinarily small amounts of liquid below pico-liter quantities (Volpatti and Yetisen, 2014). LOC systems and MS fit together remarkably well (Oedit et al., 2015).

Biosensors enable real-time detection of AFB1 in foods with a fast, sensitive, completely automated and miniaturized system (Uludag et al., 2016).

##### Flow injection immunoassays (FI-IA)

Flow injection immunoassays is an automatic method for chemical analyses, where the sample is injected into a flowing carrier solution, which is mixed with the reagents before reaching the detector. The automated system can be combined with several different detectors, e.g., biosensor, spectrophotometer, or even with mass spectrometer. For the determination of AFM1 in milk, a FI-IA method was developed with amperometric detection (Badea et al., 2014). Good potentials were demonstrated, and it was suitable as a rapid method for the screening of the toxin in raw milk. The LOD/LOQ were 0.011/0.02 ng/ml in milk with recoveries 80−120%. It should be noted that there are countries where this sensitivity of detection is not sufficient to meet the requirements of the corresponding legislation (see Table 1). Sample preparation is very simple and fast requiring only heating and dilution. Results found with this method were in good correlation with both HPLC and ELISA. The method is capable to analyze many samples in a short time. For sample preparation, the application of Protein G column is needed. The FI-IA system presented here contains low-cost devices with simple handling and it is suitable for automation (Badea et al., 2014).

### Other Techniques

Currently, several other analytical procedures are under development, which can be grouped in several ways. Some procedures are exceptions regarding the groupings as they may be allocated into more than one group such as direct analysis in real-time-mass spectrometry (DART-MS), near infrared spectroscopy (NIRS), Luminex xMAP^®^ technology and Biochip Array Technology (BAT) as a new technological direction.

#### Matrix-Assisted Laser Desorption Ionisation-Time of Flight-Mass Spectrometry (MALDI-TOF-MS)

Since there is no chromatographic or eletrophoretic separation in MALDI-TOF-MS, it is not in the group of hyphenated techniques. Ramos Catharino et al. (2005) investigated the applicability of MALDI-TOF-MS for the analysis of AFB1, AFB2, AFG1, and AFG2 content of different agricultural crops. α-Cyano-4-hydroxycinnamic acid (Et_3_N-α-CHCA) was applied as MALDI matrix and NaCl was added to the matrix in order to increase sensitivity. Even an LOD of 50 fmol could be achieved with this fast method that requires minimal sample processing. The procedure seems to be applicable for high-throughput screening not only of aflatoxins, but of other mycotoxins as well.

#### Direct Analysis in Real Time-Mass Spectrometry (DART-MS)

The DART-MS procedure includes no *de facto* separation, but the sample is usually put on a TLC or paper plate. The charged helium beam emitted from the DART ion gun is directed to the sample surface at an angle about 45°, inducing the ionization of the analyte, followed by the ESI source focusing the ionized components toward the ion entrance of the mass spectrometer (Cody et al., 2005). Busman et al. (2014) studied the possible quantitative applications of DART-MS for the aflatoxin measurement. They prepared solvent, matrix and matrix calibration standard solutions spiked with internal standard in the 1–250 ng/ml range. For all three types of calibrations, the concentration/detector response correlation was linear in the studied interval. The lowest calibration level (LCL) for AFB1 was found to be 4 μg/kg. The recovery range was 94 110%.

#### Near-Infrared Spectroscopy (NIRS)

Near-infrared spectroscopy is an innovative technology used in the food-, chemical-, pharmaceutical- and petrochemical industries. Coupled with the development of chemometric techniques, this technology is an efficient, fast, reliable and non-destructive analytical method to measure the qualitative and quantitative characteristics of organic substances. Results of earlier studies showed that the application of the NIRS technique was successful in the detection and to some extent the determination of chemical contaminants, for example mycotoxins (Tripathi and Mishra, 2009). It was observed, however, that the low sensitivity of NIR spectroscopy was not sufficient to quantitate the chemical residues in food substances. We can therefore conclude that the further development of this method is needed in order to ensure the accurate measurement of chemical contaminants found in foods and feeds. This device is able to analyze food products without any kind of preparation, but for the time being, it is considered to be quite basic for the measurement of aflatoxins (Teye et al., 2013). Because of its LOD 15–500 μg/kg, it can be used only for the prescreening of toxin-contaminated samples. More sensitive NIRS instruments are necessary for further quantitative measurements.

#### Luminex xMAP Technology

The xMAP technology enables the multiplexing of biological tests, and the reduction of time, human resources and costs spent, compared to traditional methods such as ELISA, Western blot or PCR techniques (Luminex, Austin, TX, United States). Microbeads are labeled with a special mixture of dyes, resulting in color-coded microbeads. The different microbead clusters can be mixed. As each microbead carries an individual recognition signal, the xMAP system can detect which microbead belongs to which cluster. With the aid of several lasers or LEDs, a high-speed digital signal processing system reads the processes taking place on the surface of each color-marked microbead. Red laser excites both the red and infrared dyes found in the microbeads, enabling the grouping of the microbead into one of the potential 100 clusters. Green laser induces fluorophore linked to the surface of the microbeads, enabling the determination of the substance contained in the sample. Theoretically, 100 different measurements can be performed in one sample at the same time. Peters et al. (2011) spiked 4 blank feed samples with AFB1 at the 7–23 μg/kg range with inhibition above 90−98% in all samples.

#### Fiber-Optic (Immuno)Sensor

Maragos and Thompson (1999) investigated fumonisines and aflatoxins with the fiber-optic immunosensor technique in spiked and naturally contaminated maize samples. In contrast with fumonisines, in the case of AFB1, a non-competitive sensor was used. As the fluorescence of AFB1 itself was detectable, the reaction of the sensor was proportional to the concentration of the toxin. The sensor, though could detect 2 μg/kg AFB1 in the solution, was technically not an immunosensor, as the binding of aflatoxin specific antibodies was not necessary. Therefore, this technique is not considered to be an immunochemical test. The applied sensor types are able to rapidly screen the different maize samples, but to achieve real efficiency, the sample needs to be cleaned in a separate preliminary step.

#### Biochip Array Technology (BAT)

**FIGURE 6:**
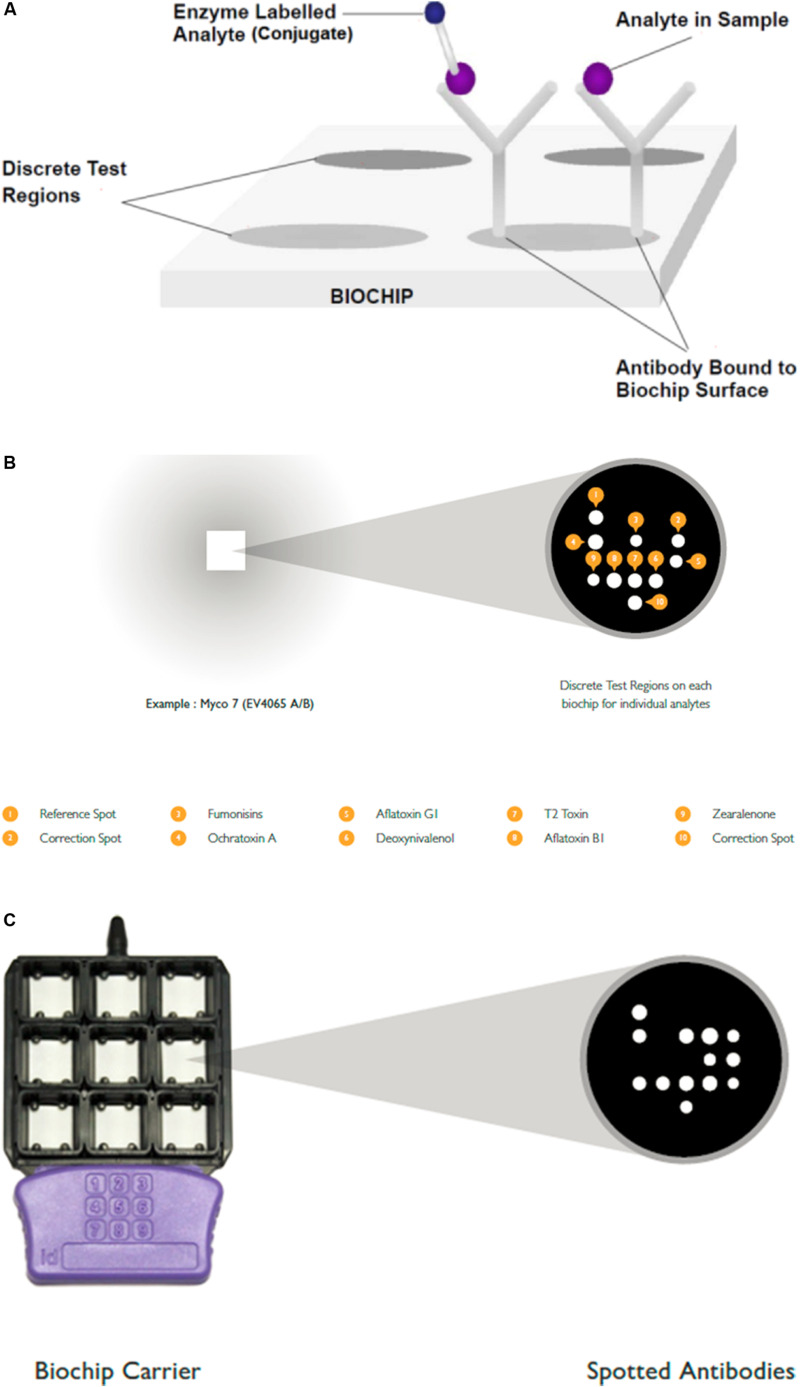
**(A–C)** shows the principle of Competitive Biochip Assay. Published with the permission of the manufacturer.

Biochip Array technology is an immunoassay based technology enabling the simultaneous semi-quantitative detection of various mycotoxins from various cereals and cereal based products. The immunoassays define discrete test regions on the biochip surface on which the immunoreactions take place. Applying specific Myco 7 kit, the screening decision levels were for aflatoxin B1 and ochratoxin A (0.25 μg/kg); aflatoxin G1, deoxynivalenol, zearalenone, T2-toxin, fumonisin B1 0.5, 100, 2.5, 5, and 10 μg/kg, respectively. The within laboratory reproducibility was 11.6% and the overall average recovery was 104%. With multiplex Myco arrays, results can be obtained within 3 h, which is comparable to that required when using a single ELISA kit. The chemiluminescence reactions can be monitored with digital picture imaging technology. such as Evidence Investigator. The flexibility of the technology allows extension of analytical profile and implementation of new assays. It should be noted that the cost of the instrument is in the range of HPLC systems, though its operation cost is lower (Figures 6A–C).

## Discussion

As aflatoxins pose danger to both humans and animals, researchers are continuously searching for analytical methods most suitable for specific tasks. Due to the development of analytical and IT techniques, increasingly faster and more sensitive have come into focus in the last decades, but only a few of them have gained applicability in routine analysis.

Immunoanalytical methods (e.g., LFIA, ELISA) proved to be promising to detect the aflatoxin present in low levels in feed and food. Immunoanalyses with portable devices are simple, fast, sensitive, and cost-effective. Occasionally they are even capable of quantification with the aid of a reader. However, application of these methods provides only informative data on the given analyzed product. Their disadvantage is that despite their general suitability for the analysis of raw materials, interferences may occur at the measurement of more complex matrices. Therefore, the areas of future research are primarily including the removal or compensation of matrix components or compensating their adverse effects, application of nanoparticle technology, specific antibody production, automation and the miniaturization of instruments.

Several immunological methods including ELISA and other fast antibody-based tests can be used for screening purposes. However, confirmatory analyses with more robust methods are needed in these cases as well.

Analytical methods for the accurate quantitative determination of aflatoxins are under constant development. Supplementary Table 1 provides guidance on the current performance characteristics of various detection techniques and highlights their limitations for practical use. Among the traditional techniques IAC clean-up followed by HPLC/FLD is the most frequently applied combination of methods for the measurement of aflatoxins. It is an excellent technique for routine laboratory analyses to comply with legal limits. Multi-mycotoxin environments (simultaneous occurrence of several mycotoxins) provide a more serious and complex health risk and challenge. Therefore, wider and more extensive monitoring of multi-mycotoxin contaminations has become necessary. At the same time, based on publications of past years reporting mycotoxin co-infections, demand for multiplex analyses is obviously rising. LC-MS/MS is an accurate and highly sensitive technique to analyze multi mycotoxins at present and years to come. It is capable to determine several mycotoxins simultaneously, and now it is considered to be a routine method. Its disadvantage is that it is an expensive technique. The operation and service costs of the instrument can be several orders of magnitude higher than those of classic LC systems. Furthermore, the treatment and maintenance of these instrument systems require a well-trained staff.

Future developments will be directed to lab-on-a-chip miniaturized technologies, chip-based biosensors and multitoxin detection by immuno-based techniques, where some analytical steps will be partly or fully replaced by micro/nanotechnology. An important goal for the research of chip-based technology is to achieve simple, fast and cost-effective methods, which can be combined with other devices and methods (e.g., immunochemical analyses) in a flexible way. It can be expected that methods and technologies, recently or further developed, will be more user-friendly and will provide better results.

Nowadays, ELISA is the most commonly used fast method in the laboratories. Using test strips for solid matrices in the fields is a technology which needs to be developed before practical application. There are many publications regarding this topic. Sample homogenization and extraction needs more development. Under industrial laboratory circumstances, methods based on test strips are mainly used as they provide faster results than ELISA.

For the confirmation of screening methods and the exact quantitative determination of aflatoxins, HPLC-FLD, combined with pre- or post-column derivatization is still the most commonly used procedure.

The best method for the exact, reproducible, qualitative and quantitative determination of aflatoxins today is HPLC-MS-MS technique using triple quadrupole mass analyzer.

However, in industrial and smaller laboratory circumstances, regarding screening tests the future is pointing toward fast and micro methods with low solvent-need, such as immuno flow cytometry.

This publication summarizes the analytical techniques that were or can be used for aflatoxin measurement or detection. The major deficiency of the majority of published methods is that they do not include the processes applied for reduction of large laboratory samples to the few grams of test portions to be extracted. Moreover, the evaluation of repeatability or reproducibility of the results, if reported, was based on a few spiked samples. Materials contaminated naturally have rarely been used to evaluate the performance of the developed methods. Much more attention is needed in the future to characterize the contribution of sample size reduction and test portion size to the overall uncertainty of the results, which are required for the correct interpretation of the measured concentration in relation to the legal limits and estimating the exposure of consumers.

In the future, when methods are evaluated from technical point of view, sources of errors must be indicated, and potential limitations of the performance parameters must be pointed out. The spike levels and the number of replicates applied must be indicated together with the reported repeatability and if possible reproducibility data. Finally, it is a must to indicate, whether repeatability and or reproducibility of mycotoxin concentration was investigated in naturally contaminated samples or not.

## Author Contributions

GM and TB contributed to create the conception and design of the review. CA, AN, and VK organized the database. GM and TB wrote the first draft of the manuscript. ÁA reviewed the first and revised drafts of the manuscript. ZF, AZ, KK, and ÁJ have done the language verification. GM, TB, and ÁA finalized the manuscript and prepared for publication. All authors contributed to manuscript revision, read, and approved the version to be submitted.

## Conflict of Interest

CA and TB were employed by Fumizol Ltd. The remaining authors declare that the research was conducted in the absence of any commercial or financial relationships that could be construed as a potential conflict of interest.
