# Corrigendum: Detection of Aflatoxins in Different Matrices and Food-Chain Positions

**DOI:** 10.3389/fmicb.2021.669714

**Published:** 2021-05-13

**Authors:** Gabriella Miklós, Cserne Angeli, Árpád Ambrus, Attila Nagy, Valéria Kardos, Andrea Zentai, Kata Kerekes, Zsuzsa Farkas, Ákos Józwiak, Tibor Bartók

**Affiliations:** ^1^Székesfehérvár Regional Food Chain Laboratory, National Food Chain Safety Office, Székesfehérvár, Hungary; ^2^Fumizol Ltd., Szeged, Hungary; ^3^University of Debrecen Doctoral School of Nutrition and Food Sciences, Debrecen, Hungary; ^4^Food Chain Safety Laboratory Directorate, National Food Chain Safety Office, Budapest, Hungary; ^5^System Management and Supervision Directorate, National Food Chain Safety Office, Budapest, Hungary; ^6^Digital Food Institute, University of Veterinary Medicine Budapest, Budapest, Hungary

**Keywords:** aflatoxins, LOD, LOQ, limits, extraction, clean-up, analysis, detection

In the original article, there was a mistake in Figure 2 as published. In the figure caption, the word “semantic” should be replaced by “schematic.” Additionally, Figure 2 has been corrected and replaced. The corrected figure and caption appear below.

**Figure 2:**
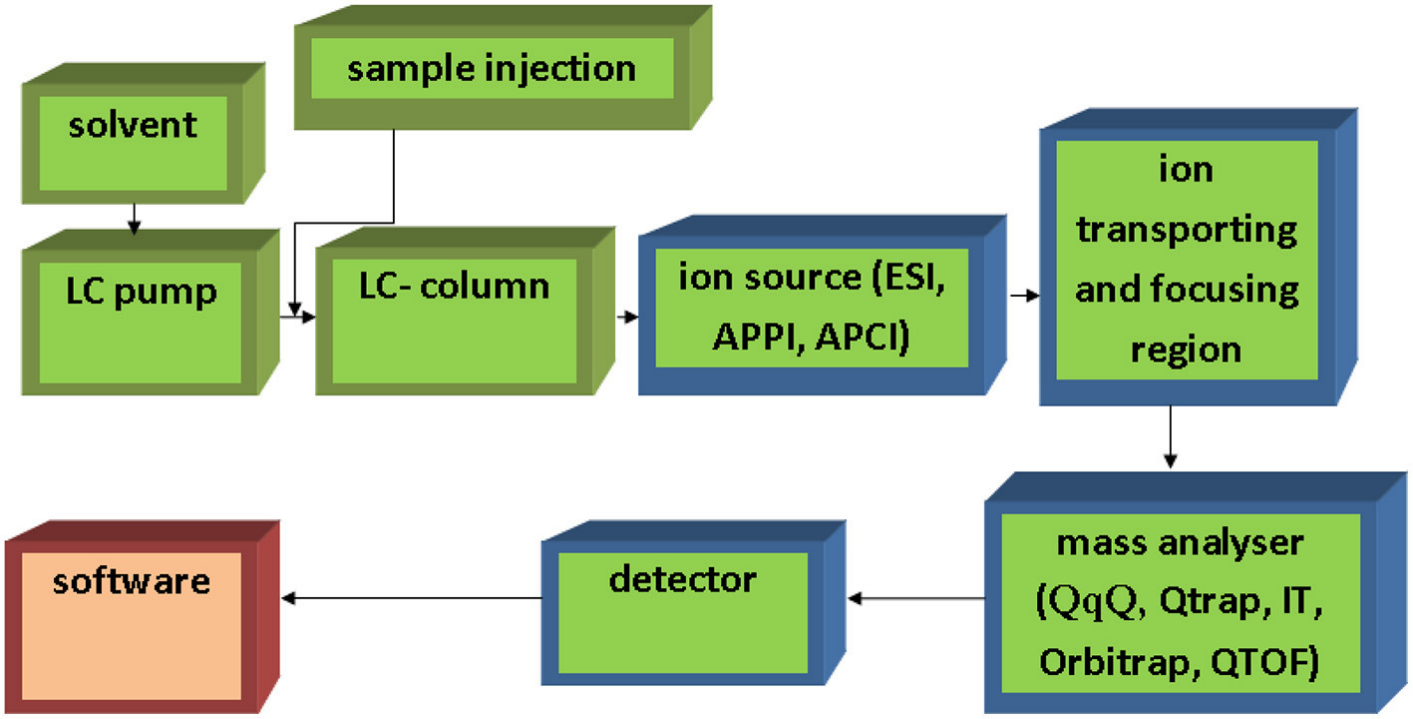
Simple schematic of LC-MS system.

In the original article, there was an error in the section heading “Chromatin Interacting Protein-Mass Spectrometry (Chip-MS).” Instead of “Chromatin Interacting Protein-Mass Spectrometry (Chip-MS)” it should be “Chip-MS.” A correction has been made to *Hyphenated Techniques, Chromatin Interacting Protein-Mass Spectrometry (Chip-MS)*.

Additionally, in the original article, there was an error in the section heading “Matrix-Assisted Laser Desorption Ionization-Time of Flight-Mass Spectroscopy (MALDI-TOF-MS).” Instead of “Matrix-Assisted Laser Desorption Ionization-Time of Flight-Mass Spectroscopy (MALDI-TOF-MS)” it should be

“Matrix-Assisted Laser Desorption Ionisation-Time of Flight-Mass Spectrometry (MALDI-TOF-MS).” A correction has been made to *Other Techniques, Matrix-Assisted Laser Desorption Ionization-Time of Flight-Mass Spectroscopy (MALDI-TOF-MS)*.

The authors apologize for these errors and state that they do not change the scientific conclusions of the article in any way. The original article has been updated.

