# Supplementary material for: Detection of Aflatoxins in Different Matrices and Food-Chain Positions
Source: Front Microbiol. 2020 Aug 14;11:1916. doi: 10.3389/fmicb.2020.01916 (PMC7480073; doi:10.3389/fmicb.2020.01916)
Supplement: Supplementary file 1 [file Data_Sheet_1.PDF]

Supplementary Material

1 Supplementary Table

Table 2. Summary of performance characteristics of detection methods

Separation techniques	Mátrix	LOD[1] (µg/kg)	LOQ[1] (µg/kg)	Recovery %	Advantages	Disadvantage	References
TLC, HPTLC	paprika, fish, maize, wheat,	0.5	-	-	Inexpensive, easy to use.	Suitable only for semi-quantitative determination at relatively high analyte concentrations; rarely used at present.	Shephard, 2009
	milk	-	0.001*	94-98			Scussel, 2003
OPLC	wheat #	0.018	0.027	84.55-105.77	It combines the advantages of HPLC, HPTLC.	No practical experience for its use.	Papp et al., 2000
HPLC/UHPLC	baby food (infant formula)#	0.02	0.1	92-101	In combination with FLD, it is the most frequently used technique in routine laboratories. Derivatives formed with PHRED are stable and provide acceptable sensitivity.	It can only be applied by experience staff under laboratory conditions. Preparation of bromine or iodine derivatives requires compliance with strict safety provisions.	Stroka et al., 2001; Gilbert and Vargas, 2003
	cereal	0.004	0.015	77.31-104.1			Rahmani et al., 2013
	milk	-	0.003*	83-108*			Yoon et al., 2016
	milk powder	0.026*	0.087*	85.4-96.9*			Wang et al., 2012
Electric driven							

techniques							
MEKC, MEKC-LIF	feed of milking cows#, alfalfa #, wheat #, bran#, maize #, grains #	0.002	0.007	70-108	These techniques can be coupled with other detection systems, such as MEKC-fiber-optic sensor (SBFOS).	There is no information for routine application.	Gao et al., 2019;
	feed#	0.02-0.06	0.12-0.42	80-130			Peña et al., 2002
Hyphenated techniques							
LC/UPLC-MS	-	-	-		none	Single MS detection does not meet selective quantification criteria. It is not applied for mycotoxin determination.	-
UPLC-ESI-QqQ-MS/MS	row milk, liquid	0.001-0.002*	0.003-0.006*	87-109*	Direct sensitive and selective detection; widely used in routine laboratories. UPLC can be coupled with QqO, QTRAP, IT, QTOF, Orbitep).	The suitable instruments are expensive. Their proper operation requires well trained experienced laboratory personnel.	Huang et al., 2014
	licorice#	0.007	0.02	72.7-123.3			Wei et al., 2013
	peanut, maize, wheat	0.1	0.3	95.3-103.3			Zhang et al., 2016
QTRAP®-LC-MS/MS	baby food	0.05	0.1	78			Rubert et al., 2012

LC-APPI-ITMS/MS	milk#	0.001*	0.006*	92-98*	Direct sensitive and selective detection; widely used in routine laboratories. UPLC can be coupled with QqO, QTRAP, IT, QTOF, Orbitep).	The suitable instruments are expensive. Their proper operation requires well trained experienced laboratory personnel.	Cavaliere et al., 2006
LC-ESI-ITMS/MS	maize #	0.6	-	79			Lattanzio et al., 2007
SFC-MS	oil	0.02	0.5	98	Ten times faster than separation with HPL; environmental friendly operation without organic solvents.	Very expensive, lack of practical experience in its use.	Lei et al., 2016
Chip-MS	peanut	0.004	0.048	99-104.9	Low solvent need for detection.	High price and operation cost. Applicable by experience staff under laboratory conditions.	Liu et al., 2013
Rapid test methods							
ELISA	row milk#	0.018*	0.025*	80-120	Simple and quick sample preparation, easy to use with simple instruments, particularly good for screening.	It is generally applied for a single analyte.	Romer Labs, Inc., Newark, USA
RIA	maize, soybean, wheat, rice	0.2	0.5	92-107	Very sensitive.	Requires expensive specific instrument and suitable radiation protection.	Korde et al., 2003
FIA, TRFIA	szójaszósz	0.1	0.3	87.2-113.3	Quick; TRFIA is suitable for selective and sensitive determination of aflatoxins.	It has limited use experience.	Wang et al., 2016
	feed samples	0.1	-	93.71-97.80			Hu et al., 2018
CFIA	maize	5	-	-	Suitable for multi mycotoxin detection; Application is simple.	Expensive instrument, the technique is under development, sensitivity is low.	Czéh, 2012, 2014; Bánáti et al.,2017
CLIA	agricultural	0.01	0.05	79.8-115.4	Automated test with low turnaround time.	Mainly used in clinical laboratories.	Fang et al., 2011

	products						
Other rapid test methods							
LFIA, LFD	food samples#	0.1 (visual)	-	-	Simple and fast to use, inexpensive, does not need specific knowledge.	Specific detection unit is needed for quantitative results; quantitative results can be obtained with specific detection units.	Liao and Li, 2010
CTRFIA	maize #	0.06	-	80.5-116.7	Simple and fast to use, inexpensive, does not need specific knowledge.	Specific detection unit is needed for quantitative results.	Zhang et. al., 2015
	peanut #	0.09	-				
	vegetable oil#	0.09	-				
	milk	0.03*	0.1*	80-110*			Tang et al., 2015
	maize	0.05	0.13	72.6-106.6			Tang et al., 2017
Biosensors (MIP)	infant food	0.0275	-	83.51-90.03	It can be integrated into a physico-chemical transducers, selective, quick detection process can be automated, inexpensive.	Equipment to be used is in experimental stage.	Semong and Batlokwa, 2017
Biosensors (OWLS)	wheat, barley, pepper	0.0005	0.001	76.4-108.6	Suitable for real time monitoring of molecular reactions.	Requires specific instrument. No routine use for AF determination.	Adányi, 2013
FI-IA	milk#	0.011*	0.020*	80-120*	Simple, quick can be coupled with various detection techniques.	No routine use for AF determination.	Badea et al., 2014
Other techniques							
MALDI-	peanut	50fmol	-	-	Simple, quick, selective, high accuracy	Due to very high price it is	Ramos

TOF-MS	s, corn, rice				m/z detection.	mainly used for research purposes.	Catharino et al., 2005
DART-MS	corn	-	4	94-110	Sample can be collected from various surface.	Less sensitive than currently used methods (e.g. HPLV/FLD).	Busman et al., 2014
NIRS	paprika, chili powder#	15	-	-	Quck, simple, suitable for multi-toxin determination.	Not sensitive for deection of food contaminants.	Teye et al., 2013
Luminex xMAP®	feed samples#	5	7	90-98	Simple, quick applicable by untrained staff.	Instrument cost is comparable to HPLC, sensitivity is low.	Peters et al., 2011
Fiber-optic (immuno)sensor	maize #	2	-	-	Quick, simple, sensitive.	Limited use in AF analysis.	Maragos and Thomson ,1999
BAT	cereals #, cereals based feed#	0.25	-	71-107	Simple, quick applicable by untrained staff, suitable for multi toxin detection.	Instrument cost is comparable to HPLC.	Plotan et al., 2016; Freitas et al., 2019

[1]: The reported values refer to AFB1, the LOD, LOQ and recovery values for AFM1 are indicated with *

[2]: The advantages and disadvantages of the methods are primarily evaluated based on the performance parameters reported by the authors of the publication. The experience of the authors served as secondary source.

#: The method was also tested with naturally contaminated samples.
